# Sternoclavicular Joint Septic Arthritis Following Blunt Trauma: Successful Surgical Management and One-Year Outcome

**DOI:** 10.7759/cureus.94458

**Published:** 2025-10-13

**Authors:** Kamil R Jarjess, John E Tobia, Saif L Juma, Jamil Haddad, Matthew J Yousif

**Affiliations:** 1 Department of Biological Sciences, College of Arts and Sciences, Oakland University, Rochester, USA; 2 Department of Natural Science, Michigan State University, East Lansing, USA; 3 Department of Osteopathic Medicine, Michigan State University College of Osteopathic Medicine, East Lansing, USA; 4 Department of Orthopedic Surgery, McLaren Macomb Hospital, Mount Clemens, USA; 5 Department of Orthopedics, Michigan State University College of Osteopathic Medicine, East Lansing, USA; 6 Department of Orthopedic Surgery, Corewell Health Beaumont Hospital, Royal Oak, USA

**Keywords:** excisional debridement, incision and drainage, mssa sternoclavicular septic arthritis, sternoclavicular joint (scj), sternoclavicular joint (scj) septic arthritis

## Abstract

We report a case of sternoclavicular joint (SCJ) septic arthritis in a previously healthy non-smoker 63-year-old male following a direct blunt impact to the anterior chest, which served as the only identifiable predisposing factor. The patient presented with a two-month history of left SCJ pain, swelling, and a chronic draining sinus tract. Laboratory findings demonstrated leukocytosis and elevated inflammatory markers, while aspiration cultures grew methicillin-sensitive *Staphylococcus aureus*. Surgical management included irrigation and debridement of the SCJ with saucerization of the necrotic medial clavicle and sinus tract excision with fat pad flap closure of the SCJ, saucerization of the necrotic medial clavicle, and excision of the sinus tract with fat pad flap closure, followed by two months of intravenous antibiotics via peripherally inserted central catheter. Pathology revealed chronic active inflammation without acute osteomyelitis. The postoperative course was unremarkable, with normalization of inflammatory markers and complete wound healing. At the one-year follow-up, the patient demonstrated a painless full range of motion with no recurrence of infection. This case highlights an uncommon presentation of SCJ septic arthritis associated with trauma and showcases the importance of early recognition, surgical intervention, and multidisciplinary management in achieving durable outcomes.

## Introduction

The sternoclavicular joint (SCJ) is a synovial saddle-like joint with an intra-articular disc composed of fibrocartilage lining [1,2]. The SCJ’s tight, poorly distensible capsule can generate high intra-articular pressures, which can promote dissemination of infection into surrounding tissues [2,3]. Furthermore, due to the SCJ’s intimate posterior relationship with the great vessels of the mediastinum, including the brachiocephalic vein, an infection can lead to devastating complications such as septic thrombophlebitis or mediastinitis [2,4,5].

SCJ infection is a rare condition, accounting for less than 1% of all septic arthritis cases [6]. It occurs predominantly in male patients presenting with chest pain. It is associated with a variety of risk factors such as diabetes mellitus, immunosuppression, intravenous drug use, and traumatic injury to the SCJ [2,4,6]. Blunt trauma to the chest wall can introduce infection directly or potentially exacerbate a transient, subclinical bacteremia [7]. However, as the sole identifiable etiology without systemic risk factors, it is rare, accounting for only ~12% of cases [1-3].

In order to prevent the spread of infection into the pleural space, mediastinum, and the great vessels, early recognition and swift treatment of SCJ infection is vital [8]. When treating SCJ infections, the literature reports that approximately 85% of all cases are managed with en bloc resection, 10% with debridement, and 5% with incision and drainage [7]. However, due to the nature of risk factors such as chest facets and wound events associated with invasive surgical procedures, minimally invasive surgical approaches should always be considered for patients with SCJ infection [7,9]. The SCJ joint's proximity to the great vessels of the mediastinum, such as the brachiocephalic vein and the superior vena cava, makes surgical management challenging and proves why posterior SCJ dislocations are dangerous [10,11]. Following broad-spectrum antibiotic therapy and diagnostic testing showing early stages of infection, simple incision and drainage represent one form of minimally invasive management in cases with no evidence of osteomyelitis or mediastinal involvement [6,7,12]. In comparison to incision and drainage, debridement remains a controversial treatment option for SCJ infection due to its higher recurrence rates and risks of persistent infection, requiring further surgical intervention [13].

We report the case of a non-smoker 63-year-old male, who developed an SCJ infection following blunt chest trauma, with no systemic risk factors identified, and was successfully treated with incision and drainage.

## Case presentation

A 63-year-old male patient presented to the clinic with a two-month history of progressive pain, erythema, swelling, and a chronic draining sinus tract over the left sternoclavicular (SC) joint. He reported a history of blunt trauma to the joint approximately two months prior to presentation but denied intravenous drug use, recent systemic infection, or other known risk factors for septic arthritis. His medical history was prominent for a deep vein thrombosis with inferior vena cava filter placement 15 years before presentation to the clinic.

**Figure 1:**
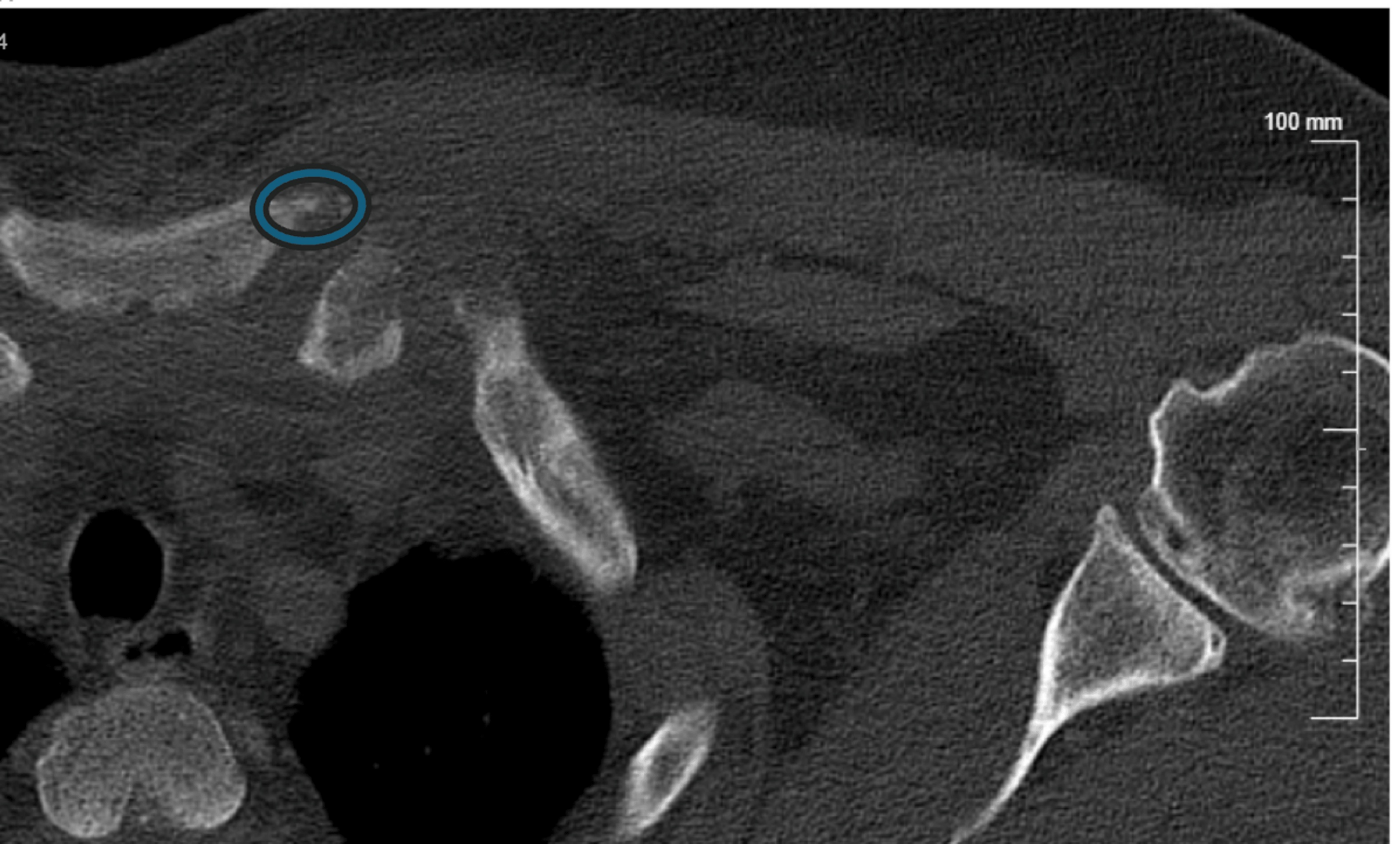
CT scan (bone window) The axial CT scan with bone window at the level of the sternoclavicular joint demonstrates cortical irregularity and erosive changes involving the medial (sternal) end of the left clavicle. The blue circled region highlights cortical disruption and periarticular erosion on the clavicular side, with adjacent soft tissue swelling suggestive of inflammatory extension beyond the joint.

**Figure 2:**
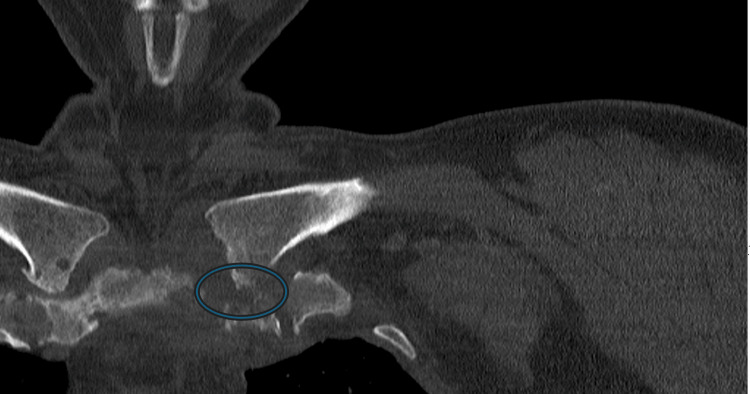
CT scan (coronal view) The coronal CT reveals asymmetric widening of the left sternoclavicular joint compared to the contralateral side. The blue circled region highlights osseous destruction at the medial clavicle and adjacent manubrial articulation. Surrounding soft tissue density changes suggest an infectious or inflammatory process, possibly with phlegmon formation.

Preoperative CT images of the SCJ were obtained, which demonstrated erosive changes at the medial clavicle with cortical destruction and irregular joint margins. The axial cuts revealed surrounding soft tissue swelling consistent with inflammatory extension, while the coronal view confirmed asymmetric joint widening with bony erosion of both the clavicular and sternal sides of the articulation (Figures 1, 2). These findings supported the diagnosis of septic arthritis of the SC joint with adjacent osseous involvement, prompting the decision for operative intervention.

Initial laboratory results obtained by the infectious disease (ID) service revealed leukocytosis (WBC 12.7 × 10³/µL; normal: 4.5-11.0 × 10³/µL), thrombocytosis (platelets 578 × 10³/µL; normal: 150-450 × 10³/µL), elevated C-reactive protein (172 mg/L; normal: <5 mg/L), and an erythrocyte sedimentation rate (ESR) of 105 mm/hr (normal: 0-20 mm/hr in males) [13-15] (Table 1). Ultrasound-guided aspiration of the SC joint showed methicillin-sensitive *Staphylococcus aureus* (MSSA). Given persistent symptoms and continued drainage despite initial management, surgical exploration was recommended.

**Table 1:** Initial laboratory results with adult reference ranges (male) Reference ranges may vary by laboratory; values shown reflect commonly accepted adult ranges from StatPearls (CBC) [13], Mayo Clinic Laboratories (CRP) [14], and StatPearls (ESR) [15]. *ESR reference intervals are age- and sex-specific. CBC, complete blood count; CRP, C-reactive protein; ESR, erythrocyte sedimentation rate

Test	Patient value	Reference range (adult male)	Interpretation
White blood cell count (×10³/µL)	12.7	4.5–11.0	High
Platelet count (×10³/µL)	578	150–400	High
CRP (mg/L)	172	<5	High
ESR (mm/hr)*	105	≤20 (men >50 y)	High

**Figure 3:**
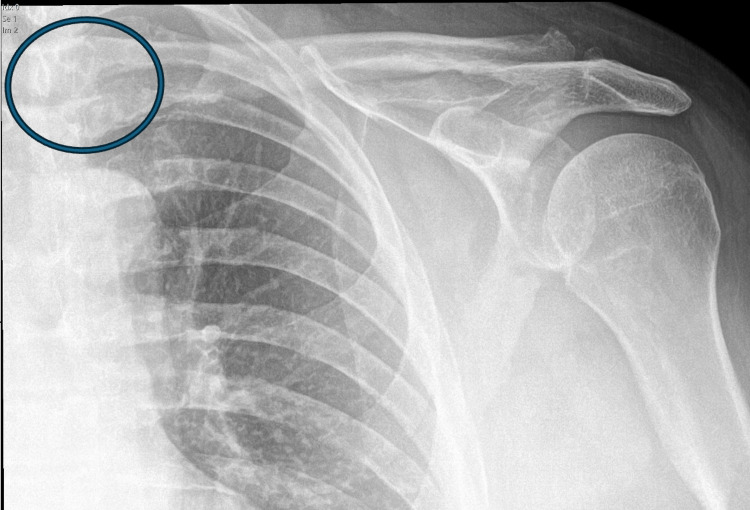
Postoperative anteroposterior radiograph of the left shoulder Anteroposterior radiograph of the left shoulder demonstrating resection of the medial (sternal) end of the clavicle (circled in blue). Joint alignment is preserved with no evidence of residual dislocation or acute fracture.

The patient underwent irrigation and debridement of the left SC joint, saucerization of the necrotic medial clavicle, closed reduction and manipulation under anesthesia, and excision of the chronic draining sinus tract with fat pad flap closure. Multiple intraoperative specimens, including bone and soft tissue, were collected and sent for histopathology and microbiological cultures (anaerobic, fungal, acid-fast bacillus, and wound cultures) before administration of antibiotics. Clindamycin 600 mg IV was administered intraoperatively after specimen collection. A 6-cm midline incision over the SC joint was made, the chronic draining sinus tract was excised, and a fat pad flap was fashioned for closure. Saucerization of the necrotic medial clavicle was performed. No drains or implants were placed. Intraoperatively, there was no gross purulence, but the distal clavicle demonstrated necrotic changes. Postoperative imaging revealed no acute fracture, dislocation, or residual subluxation (Figure 3).

Pathology findings

Gross examination of the surgical specimen revealed a 2.5 × 1 × 0.3 cm aggregate of hard tan-red bone fragments mixed with focal tan-pink soft tissue. Microscopic analysis demonstrated fibrocartilaginous tissue with chronic active inflammation and fragments of bone with hemorrhage and reactive changes. No definitive features of acute osteomyelitis were identified.

At postoperative week 1, cultures from the operative site grew moderate *Staphylococcus aureus* and a few gram-positive cocci in clusters with rare polymorphonuclear leukocytes. The patient was admitted for intravenous antibiotics, and a peripherally inserted central catheter (PICC) line was placed for prolonged therapy under ID guidance. ID service recommended cefazolin 2 g IV every 8 hours, which was continued for approximately two months. The PICC line was removed approximately two months later upon completion of the antibiotic course.

At the time of follow-up, the patient demonstrated complete wound healing, resolution of pain and swelling, and a return to baseline function without recurrence of infection. At one year postoperatively, clinical evaluation revealed a painless, anterior, reducible subluxation with a painless, symmetrical, and full range of motion of the left SCJ with a Constant-Murley score of 83.

## Discussion

SCJ infections are rare, accounting for less than 1% of all septic arthritis cases [6]. They are most often associated with systemic risk factors such as intravenous drug use, diabetes mellitus, immunosuppression, and indwelling central venous catheters [2,6]. In contrast, our patient’s only notable history was localized trauma two months prior to symptoms onset, demonstrating that even in the absence of systemic risk factors, mechanical injury may predispose to infection.

This case represents a delayed presentation rather than a delayed diagnosis, as the patient did not seek medical attention until two months after the onset of symptoms. The prolonged, untreated course likely contributed to the chronic features observed, including necrotic changes of the medial clavicle and adjacent osteitis, as evident on imaging. Interestingly, histopathology revealed chronic inflammation without acute osteomyelitis, showcasing the variable pathological spectrum of SCJ infections.

Typical presenting complaints in SCJ infection include localized pain and swelling over the joint, often accompanied by erythema and limited range of motion of the upper extremity due to pain, and some patients might experience fever, malaise, or constitutional symptoms [2]. Early symptoms can mimic other musculoskeletal conditions of the upper chest or shoulder girdle, leading to a delay in diagnosis due to a low index of suspicion [2].

*Staphylococcus aureus*, particularly methicillin-sensitive strains (MSSA), remains the most common pathogen isolated in SCJ infections [16]. Our patient’s cultures confirmed MSSA both preoperatively and in the early postoperative period. The presence of a chronic draining sinus tract and necrotic clavicular bone without histological evidence of acute osteomyelitis is unusual. This may represent a contained, chronic infective process where the immune response had walled off the infection, leading to bony necrosis without the typical acute inflammatory infiltrate of osteomyelitis. It may represent a transition from acute to chronic infection with partial containment by surrounding tissues.

**Figure 4:**
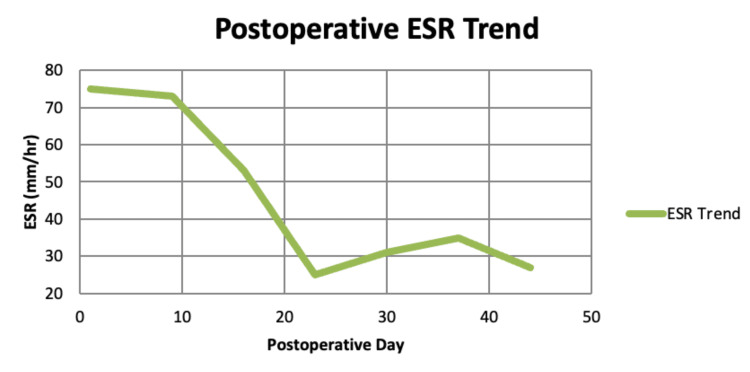
Postoperative ESR trend Postoperative ESR trend demonstrating progressive decline from 75 mm/hr on day 1 to 27 mm/hr by day 44, correlating with the patient’s clinical improvement and infection resolution. ESR, erythrocyte sedimentation rate

From a laboratory perspective, our patient demonstrated significant systemic inflammation at presentation, with leukocytosis (WBC 12.7 × 10³/µL), thrombocytosis (platelets 578 × 10³/µL), elevated C-reactive protein (172 mg/L), and an ESR of 105 mm/hr. Postoperatively, inflammatory markers were closely monitored, showing a progressive decline in ESR: 75 mm/hr (postoperative day 1), 73 mm/hr (postoperative day 9), 53 mm/hr (postoperative day 16), 25 mm/hr (postoperative day 23), 31 mm/hr (postoperative day 30), 35 mm/hr (postoperative day 37), and 27 mm/hr (postoperative day 44) (Figure 4). This downward trend paralleled the patient’s clinical improvement, supporting effective infection control and resolution of the inflammatory process.

Management of SCJ infections generally requires a combined approach of surgical intervention and targeted antibiotic therapy [2]. While the primary goal of surgery was infection control, the extensive debridement and bony resection create a local inflammatory environment that, in rare cases, can predispose to heterotopic ossification as a delayed complication. This process of aberrant bone formation has been documented in various soft tissues following trauma or surgical intervention, including highly atypical locations [17], making postoperative vigilance necessary to monitor for this potential sequela. This approach of treatment aligns with the literature emphasizing aggressive local control to prevent the recurrence of SCJ infection [16,18]. In one of the most extensive institutional series, Ali et al. reported recurrence rates as low as 2.3% overall, with flap-assisted closures achieving the most durable results [18]. Similarly, Kachala et al. found an approximate 10% recurrence rate after surgical management, with the majority of recurrences occurring in the early postoperative course [19]. Our patient’s successful outcome following debridement and flap closure aligns with reports suggesting that, in cases without mediastinal extension or extensive osteomyelitis, debridement can achieve comparable infection control to en bloc resection while minimizing morbidity [2,7].

## Conclusions

We reported a SCJ infection in a 63-year-old man following blunt chest trauma without systemic risk factors. Operative irrigation and debridement, including saucerization and sinus-tract excision, along with fat pad flap closure and prolonged cefazolin therapy, achieved durable infection control. The patient returned to baseline function with a Constant-Murley score of 83 and had no recurrence at one year. This case highlights the importance of timely recognition, focused surgery, a tailored antimicrobial plan, and coordinated multidisciplinary care.
